# Bee venom attenuates neuroinflammatory events and extends survival in amyotrophic lateral sclerosis models

**DOI:** 10.1186/1742-2094-7-69

**Published:** 2010-10-15

**Authors:** Eun Jin Yang, Jing Hua Jiang, Sang Min Lee, Sun Choel Yang, Hye Suk Hwang, Myeong Soo Lee, Sun-Mi Choi

**Affiliations:** 1 Department of Standard Research, Korea Institute of Oriental Medicine, 483 Expo-ro, Yuseong-gu, Daejeon, 305-811, Korea; 2 Department of Instrument Development, Korea Basic Science Institute, 113 Gwahag-ro, Yuseong-gu, Daejeon, 305-333, Korea

## Abstract

**Background:**

Amyotrophic lateral sclerosis (ALS) is a disease affecting the central nervous system that is either sporadic or familial origin and causing the death of motor neurons. One of the genetic factors contributing to the etiology of ALS is mutant SOD1 (mtSOD1), which induces vulnerability of motor neurons through protein misfolding, mitochondrial dysfunction, oxidative damage, cytoskeletal abnormalities, defective axonal transport, glutamate excitotoxicity, inadequate growth factor signaling, and neuroinflammation. Bee venom has been used in the practice of Oriental medicine and evidence from the literature indicates that BV plays an anti-inflammatory or anti-nociceptive role against inflammatory reactions associated with arthritis and other inflammatory diseases. The purpose of the present study was to determine whether bee venom suppresses motor neuron loss and microglial cell activation in hSOD1^G93A ^mutant mice.

**Methods:**

Bee venom (BV) was bilaterally injected (subcutaneously) into a 14-week-old (98 day old) male hSOD1^G93A ^animal model at the Zusanli (ST36) acupoint, which is known to mediate an anti-inflammatory effect. For measurement of motor activity, rotarod test was performed and survival statistics were analyzed by Kaplan-Meier survival curves. The effects of BV treatment on anti-neuroinflammation of hSOD1^G93A ^mice were assessed via immunoreactions using Iba 1 as a microglia marker and TNF-α antibody. Activation of ERK, Akt, p38 MAP Kinase (MAPK), and caspase 3 proteins was evaluated by western blotting.

**Results:**

BV-treated mutant hSOD1 transgenic mice showed a decrease in the expression levels of microglia marker and phospho-p38 MAPK in the spinal cord and brainstem. Interestingly, treatment of BV in symptomatic ALS animals improved motor activity and the median survival of the BV-treated group (139 ± 3.5 days) was 18% greater than control group (117 ± 3.1 days). Furthermore, we found that BV suppressed caspase-3 activity and blocked the defects of mitochondrial structure and cristae morphology in the lumbar spinal cord of hSOD1^G93A ^mice at the symptomatic stage.

**Conclusion:**

From these findings, our research suggests BV could be a potential therapeutic agent for anti-neuroinflammatory effects in an animal model of ALS.

## Background

Amyotrophic lateral sclerosis (ALS) is a severe paralytic disorder of the central nervous system (CNS) that is characterized by age-related degeneration or elimination of upper and lower large motor neurons of the motor cortex, brainstem, and spinal cord [1]. ALS symptoms are characterized by muscle weakness, atrophy, spasticity, and paralysis [2]. Riluzole therapy has been shown to only improve the survival of ALS patients by a few months and has several side-effects, including asthenia, nausea, anorexia, and diarrhea [3]. Therefore, a safer and more effective therapy for ALS patients is needed in order to reduce the pain associated with this disease. Most ALS patients have the sporadic form of the disease while 5-10% of ALS cases are of the familial type. Familial ALS (fALS) cases are caused by autosomal dominant mutations of the human Cu-Zn superoxide dismutase 1 (hSOD1) gene [4]. An animal model of ALS has been characterized in mice that carry the mutated hSOD1 gene with a glycine to alanine substitution at the 93^rd ^codon (SOD1^G93A^). These animals display progressive motor neuron degeneration that is similar to that observed in cases of human ALS [5]. *In vivo *and *in vitro *studies using mutant hSOD1 transgenic mice have demonstrated multiple cellular pathogenic events in motor neurons such as glutamate excitotoxicity, mitochondrial dysfunction, protein misfolding, neurofilament accumulation, and oxidative stress [2,6,7].

Neuroinflammation is a pathological feature present in ALS patients and in the mutant hSOD1 mouse model [8]. As critical mediators of inflammation, activated microglia and elevated levels of tumor necrosis factor-alpha (TNF-α) are detected in the CNS of ALS patients and in hSOD1^G93A ^transgenic mice [9]. Previous findings point towards the critical involvement of microglia in the development of motor neuron disease; however the nature of microglial-neuronal interactions that lead to motor neuron degeneration remains unknown. One possibility that has been extensively studied in the context of other neurodegenerative diseases, especially Alzheimer's disease (AD), is the notion that chronic and detrimental microglial neuroinflammation contributes to neuronal degeneration [10]. Another study demonstrated that p38, a 38 kDa Stress Activated Protein Kinase/Mitogen-Activated Protein Kinase (SAPK/MAPK), has a key role in the development of inflammation [11]. Inhibition of p38 has been shown to produce anti-inflammatory effects by reducing cytokines such as TNF-α, interleukin-1 (IL-1), and nitric oxide synthetase (NOS) as well as by decreasing Cox-2 in inflammatory cells [11]. Elevated levels of TNF-α and TNF-α medicated signaling pathways are evident in a large number of neurological disorders including multiple sclerosis (MS), AD, Parkinson's disease (PD), and ALS [12-15]. Furthermore, pro-inflammatory cytokines, such as TNF-α and interferon-gamma (IFN-γ) have been proposed to be involved in ALS-like microglial activation and inflammation-induced motor neuron death [16,17].

Bee venom (BV), which is also known as apitoxin, is extracted from honeybees and is commonly used in Oriental medicine. The anti-inflammatory action of BV has shown to reduce pain in patients with chronic rheumatoid arthritis and osteroarthritis [18]. However, the mechanism by which these anti-inflammatory properties relieve the neuroinflammation associated with neurodegenerative disease is not clear. In order to explore this therapeutic mechanism, we investigated the anti-inflammatory effects of BV on motor function and survival in SOD1^G93A ^mutant mice.

The present study showed decreased levels of TNF-α and the deactivation of p38 MAPK downstream of the TNF-α signaling pathway in both the brainstem and spinal cord of hSOD1^G93A ^mice after treatment with BV in the hind limbs at the ST36 acupoint. Interestingly, BV treatment reduced the number of microglial cells and astrocytes, and dramatically increased the expression of MAP2 in motor-related regions of the brainstem and spinal cord in comparison with age-matched hSOD1^G93A ^control mice. Additionally, we showed that BV suppressed caspase-3 activity and reduced the disruption of mitochondrial structure and cristae morphology in the lumbar spinal cord of hSOD1^G93A ^mice. Furthermore, BV treatment improved the motor coordination and prolonged the life span of familial mutant ALS animals at a symptomatic stage. Based on these findings, we suggest that BV has a neuroprotective effect against motor neuron cell death and suppresses neuroinflammation-induced disease progression in symptomatic ALS mice model.

## Methods

### Animals

All mice were handled in accordance with the guidelines of the United States National Institutes of Health (Bethesda, MD). The protocols were approved by the Institutional Animal Care and Use Committees of the Korea Institute of Oriental Medicine. Hemizygous transgenic B6SJL mice carrying a glycine to alanine base pair mutation at the 93^rd ^codon of the cytosolic Cu/Zn superoxide dismutase gene (hSOD1^G93A^) were originally obtained from the Jackson Laboratory (Bar Harbor, ME). Transgenic mice were identified by PCR as described previously [4]. The hSOD^G93A ^mice developed the initial signs of neuromuscular deficits, such as leg tremors and loss of the hindpaw extension reflex, at approximately 15 weeks of age. At 16 weeks, they showed marked locomotor impairment with paralysis and muscular atrophy of the hind limbs. These animals died due to respiratory failure at 18-20 weeks of age. The 14-week-old transgenic mice were considered to be symptomatic; mice of this age were used for the study. All mice were kept in standard housing with free access to water and standard rodent chow.

### Bee venom treatment

Bee venom was purchased from Sigma (St. Louis, MO) and diluted with saline. At a dose of 0.1 μg/g, bee venom was bilaterally injected (subcutaneously) into 14-week-old (98 day old) male hSOD1^G93A ^transgenic mice (hSOD1^G93A^-BV, n = 11) at the Zusanli (ST36) acupoint, which is known to mediate an anti-inflammatory effects [19]. BV treatment was performed two times a week at a dose of 0.1 μg/g. According to the human acupoint landmark and a mouse anatomical reference [20], the ST36 point is anatomically located at 5 mm below and lateral to the anterior tubercle of the tibia. Control animals were bilaterally injected (subcutaneously) with an equal volume of saline at the ST36 acupoint (hSOD1^G93A^, n = 11).

### Behavioral analysis (rotarod test)

Mice were trained for 1 week in order for them to acclimate to the apparatus. After training of the hSOD1^G93A ^mice, their basal motor activity was measured with a rotarod apparatus (Ugo, Basil, Italy). Motor coordination was assessed by measuring the length of time that the mice remained on the rotating rod (10 rpm) as was previously described [21].

Each animal underwent three trials and the average time spent on the rod was determined for each group. Rotarod testing was conducted at the same time to reduce environmental variables such as light cycle and temperature.

### Life span study

For lifespan analysis, SOD1^G93A ^mice were assessed for a total of 100 days and divided into the following treatment groups: saline-treated SOD1^G93A ^mice (n = 11) and SOD1^G93A ^mice treated with BV at 14 weeks (98 day old) old (n = 11). We defined "death" that is, the end point of life for hSOD1^G93A ^mice as the first day on which the mouse stopped movement on either side, after which time the mouse was no longer able to eat due to severe hindlimb paralysis. Mice were then sacrificed to reduce further pain from respiratory failure according to animal care guidelines. The life span analysis was carried out in male SOD1^G93A ^mice. Survival statistics were analyzed by Kaplan-Meier survival curves by Prism 4.0 software (GraphPad Software, San Diego, CA) and Sigmaplot 10 software (Systat Software, Inc. Richmond, CA).

Values were analyzed by a one-way ANOVA followed by a Dunn's multiple-comparison test. All statistics were performed and graphs were developed using Prism 4.0.

### Tissue processing and immunohistochemistry

At 18 days after treatment with BV or saline, hSOD1^G93A ^mice were deeply anesthetized with pentobarbital. They were transcardially perfused with phosphate-buffered saline (PBS), followed by a perfusion with a fixative solution containing 4% paraformaldehyde in PBS. The spinal cord and brain were dissected out and fixed overnight in 4% paraformaldehyde at 4°C, transferred to 30% sucrose, and then frozen. The spinal cord and brainstem were embedded in OCT compound and serially cut on a cryostat into 40-μm thick coronal sections. The sections were then floated in PBS and quenched with 30% methanol and hydrogen peroxide to eliminate endogenous peroxidase activity. After blocking in 5% normal goat serum at room temperature, sections were incubated with the primary antibodies, which included Iba-1 (Wako, Osaka, Japan) at 1:5000 or TNF-α (Cell Signal, Beverly, MA) at 1:100. All tissue sections were rinsed in PBST (PBS with 0.3% Tween 20) and incubated in secondary antibody (1:1000) for 1 h. Following incubation, all sections were rinsed and stained using Vectastain ABC kits (Vector, Burlingame, CA) according to the manufacturer's instructions. For visualization, 3, 3'-diaminobenzidine (DAB)-H_2_O_2 _substrate was used with a hematoxylin counterstain. After rinsing, all samples were dehydrated in increasing concentrations of ethanol, cleared in xylene, and coverslipped using Permount mounting medium (Fisher Scientific, Pittsburgh, PA). Immunostained tissues were observed with a light microscope (Olympus, Tokyo, Japan). Every fifth spinal cord or brainstem section (50 μm) from saline- (n = 3) or BV-treated hSOD1^G93A ^mice (n = 4) was stained with Iba 1 or TNF-α antibody. Cell counting for immunoreactive cells was performed using an image analysis software (IMT *i*-solution, Hackettstown, NJ).

### Western blot

At 14 days after treatment with BV or saline, brains and spinal cords were dissected and homogenized in RIPA buffer (50 mM Tris-Cl pH 7.4, 1% NP-40, 0.1% SDS, and 150 mM NaCl). Homogenized tissues were centrifuged at 14,000 rpm for 20 min at 4°C. Proteins were quantified using the BCA assay kit (Pierce, Rockford, IL). Samples were electrophoresed through SDS-polyacrylamide gels and transferred to nitrocellulose membranes. Blots were blocked with 5% non-fat milk in TBS for 1 h prior to incubation with antibodies. Various primary antibodies were utilized in this study, including anti-SOD1 (Calbiochem, La Jolla, CA), anti-tubulin (Abcam, Cambridge, UK), anti-Akt (Cell Signaling, Beverly, MA), anti-pAkt (Cell Signaling), anti-p38 (Cell Signaling), anti-phospho-p38 (Cell Signaling), anti-Iba 1 (Wako, Osaka, Japan), and anti-active caspase-3 (Calbiochem). Blots were probed with HRP-conjugated antibodies (SantaCruz, Santa Cruz, CA) and developed with enhanced chemiluminescence (ECL) reagents (Amersham Pharmacia, Piscataway, NJ).

### Transmission Electron Microscopy (TEM)

BV-treated hSOD1^G93A ^mice and age-matched controls were sacrificed and perfused with 2% glutaraldehyde. The procedure of sample preparation for TEM was performed as previously described [22].

### Statistical analysis

Results are expressed as means ± SEM values. Statistical evaluations were conducted using the Mann-Whitney U test for comparisons between BV-treated and age-matched untreated hSOD1^G93A ^mice. A *t*-test was used to compare the immunoblotting and immunohistochemical data between the BV-treated mice and the age-matched untreated hSOD1G93A mice. P values less than 0.05 were considered significant. All analyses were performed with SPSS 12.0 software (SPSS, Chicago, IL).

## Results

### The BV treatment increases survival rate and motor activity in symptomatic hSOD1^G93A ^transgenic mice

**Figure 1:**
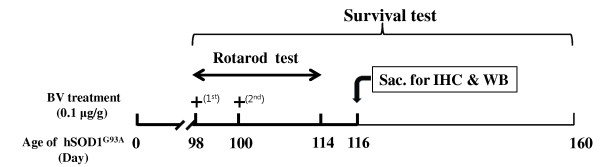
**Diagram of the experiment schedule**. At the beginning of the experiment, the animals were randomly divided into two groups: the saline-treated group (control) and the BV-treated group. Saline- or BV- (0.1 μg/g) was administrated bilaterally and subcutaneously at ST36 to 98-day-old hSOD1^G93A ^male mice (first treatment for saline or BV). Two days after the first saline or BV treatment, saline or BV (0.1 μg/g) was injected by the same method. A rotarod test for the measurement of motor activity was performed from day 91 to day 114. For the survival test, saline- and BV-treated mice were observed until they reached 160 days old. Mice used for the biochemical study were sacrificed 18 days after the first saline- or BV treatment. IHC: immunohistochemistry, WB: western blotting

**Figure 2:**
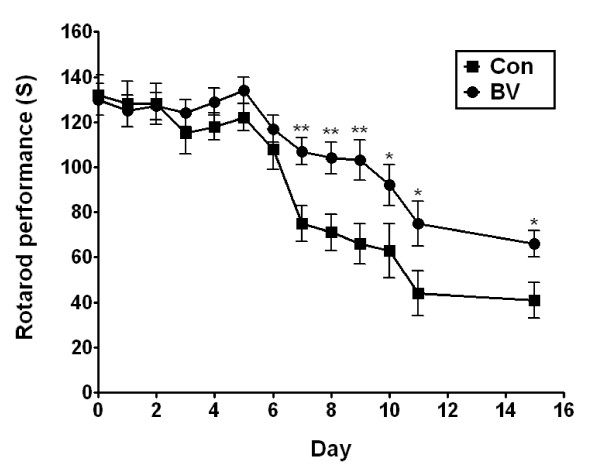
**Effects of BV on rotarod performance of hSOD1**^**G93A **^**mice**. BV delays the onset of motor impairment in hSOD1^G93A ^transgenic mice. B6 G93A-SOD1 mice (n = 11/group) were injected with saline (squares) or BV at ST36 (circles). Values represent the mean ± SEM. Significantly improved motor performance was evident at most time points between 7-9 days after BV treatment as compared with controls. The Mann-Whitney U test was used to compare saline-treatment versus BV-treatment in hSOD1^G93A ^transgenic mice (**p < 0.005, *p < 0.05). S: second, Con: saline treated-mice, BV: bee venom treated-mice.

**Figure 3:**
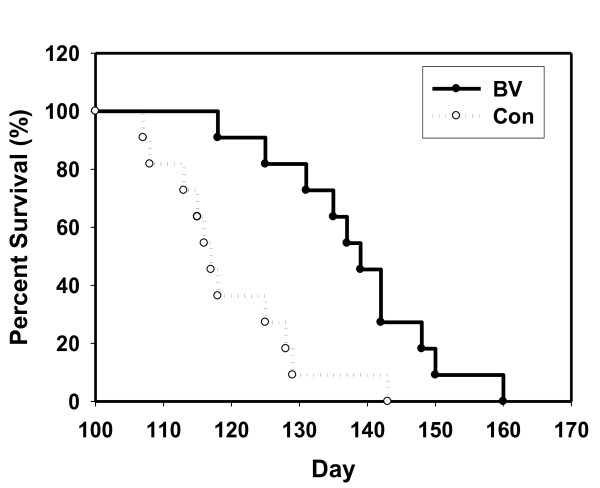
**BV prolongs the survival of hSOD1**^**G93A **^**transgenic mice**. A Kaplan-Meyer analysis illustrates the significant benefits from BV (0.1 μg/g/98day) compared with the control regarding survival rate. Mice were bilaterally injected with saline (open circles) or BV (closed circles) at ST36 (subcutaneously). The mean, median, minimum, and maximum age of death in saline-treated hSOD1^G93A ^mice are 120, 117, 107, 143 day, respectively. The mean, median, minimum, and maximum age of death in BV-treated hSOD1^G93A ^mice are 139, 139, 118, and 160 day, respectively. Con: saline-treated mice, BV: bee venom-treated mice.

To determine the effects of BV on the survival and motor activity of hSOD1^G93A ^mice, BV (0.1 μg/g) was bilaterally administered (subcutaneously) at ST36 (Figure 1). As shown in Figure 2, the BV-treated hSOD1^G93A ^group displayed a 1.3-fold increase in motor function at 7 days after treatment with BV as determined by the rotarod behavioral test compared to age-matched control mice. Furthermore, we observed that BV-treated mice had a delay in disease onset and paralysis compared to saline-treated hSOD1^G93A ^mice. Next, we examined the survival rate to determine whether BV treatment prolonged the life span of hSOD1^G93A ^mice. The expected life span was assessed by Kaplan-Meier survival analysis. The median survival of the BV-treated group (139 ± 3.5 days) was 18% greater than control group (117 ± 3.1 days). The Kaplan-Meier probability of survival analysis showed that the BV-treated group had a significantly improved survival rate (160 ± 3.5 days) compared to the control group (143 ± 3.1 days) (Figure 3). However, there was no significant difference in body weight before and after BV treatment (data not shown). These results indicate that BV acted therapeutically against the onset of motor dysfunction as well as against disease progression in hSOD1^G93A ^mice.

### BV reduces microglial cell activation and neuroinflammation

**Figure 4:**
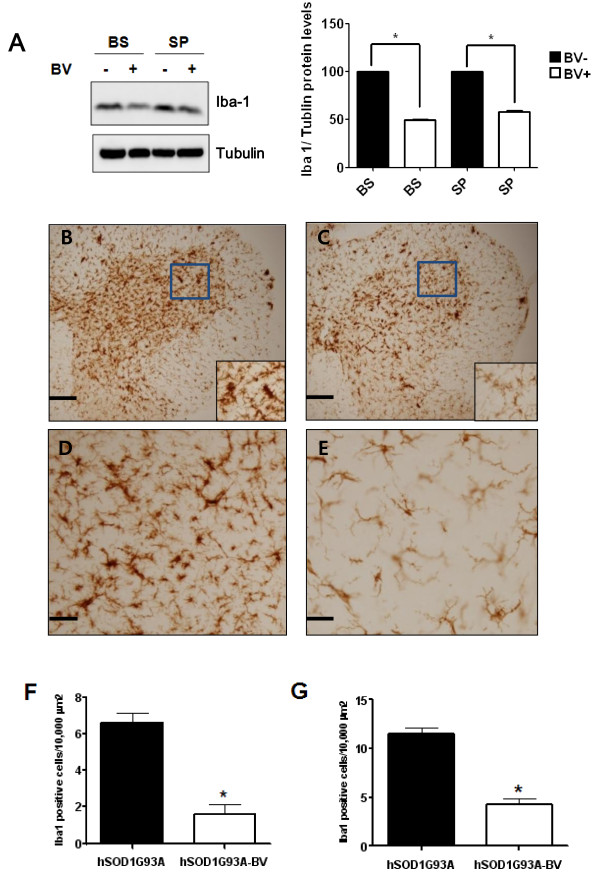
**Immunoreactivity (IR) and western blotting for Iba1 in the brainstem and spinal cord**. A representative blot of Iba-1 is shown significant reduction of Iba I in the brainstem and spinal cord of BV-treated hSOD1^G93A ^mice (A). This experiment was performed using saline- or BV-treated hSOD1 ^G93A ^mice (n = 3). Quantitative analysis of immunoblot. The image is representative of three independent experiments. The optical density was measured for each band, and values for Iba-1 were compared with tubulin after correcting for the total protein content. The Results of the densitometric quantifications are the means ± SEM of triplicate samples. The data were analyzed using a *t*-test. *p < 0.01 versus the corresponding saline-treated group. Iba1 IR of the lumbar (L4) spinal cord of hSOD1^G93A ^mice treated with saline (B) or BV (C). Boxes indicate high magnification views of the ventral horn region. Iba1 IR of the facial nucleus of the brainstem from saline- (D) or BV-treated transgenic mice (E). Scale bars = 200 μm (B, C). Scale bars = 100 μm (D, E). Cell counts of Iba1-positive cells in the ventral horn region or brainstem of saline- (black columns, n = 3) or BV-treated mice (white columns, n = 4) (F, G). Data are shown as the mean ± SEM. Data were analyzed with a *t*-test. *p < 0.001. BV: bee venom, BS: brainstem, SP: spinal cord

Microglia activation can be observed in virtually all CNS pathologies including ALS. However, the role of the microglial response in many CNS disorders remains ambiguous, given that these cells exhibit both neuroprotective and neurotoxic effects [23]. To demonstrate whether BV affected neuroinflammation in a familial ALS animal model, we studied the relationship between microglia activation and inflammatory factors in symptomatic hSOD1^G93A ^mice. For this experiment, BV or saline was injected at ST36 in 14-week-old hSOD1^G93A ^mice. The effect of BV on activated microglial cells monitored using the Iba-1 antibody in both the brainstem and lumbar spinal cord of symptomatic hSOD1^G93A ^mice. As shown in Figure 4A, the expression level of Iba-1 was dramatically reduced in both the brainstem and spinal cord of BV-treated mice in comparison with the control group. Next, we immunostained lumbar spinal cord sections from BV- or saline-treated mice (Figure 4B-G). At a low magnification, Iba-1 immunoreactivity was detected in both the white and gray matter of the spinal cord in hSOD1^G93A ^transgenic mice (Figure 4B,C). At a higher magnification, Iba-1 immunoreactivity in the ventral horn of BV-treated hSOD1^G93A ^mice was significantly reduced (~2.8 times) as compared to the identical area in control mice (Figure 4F). In the brainstem of mutant SOD1 mice, Iba-1 immunoreactive cells were detected as well (Figure 4D,E). BV caused an approximate 2.4 fold decrease in microglial activity within the facial nucleus of the brainstem (Figure 4G).

**Figure 5:**
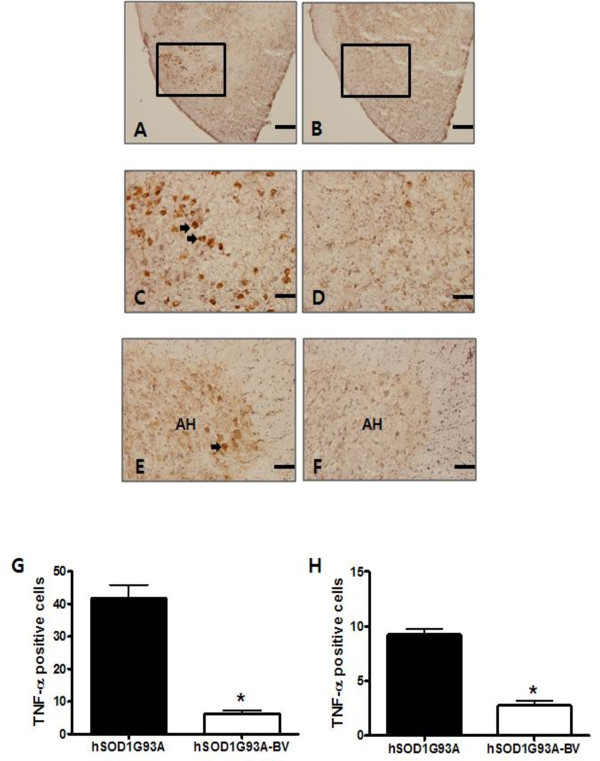
**Immunohistochemical study of TNF-α in the brainstem and anterior horns of the lumbar (L4) spinal cord in BV- or saline-treated familial mutant SOD1 mice**. TNF-α IR is significantly reduced in the facial nucleus of the brainstem from BV-treated hSOD1^G93A ^mice (A, B). Scale bars = 400 μm (A, B). High magnification of boxes (facial nucleus) in A and B (C, D). In the anterior horn of the spinal cord, the number of TNF-α-immunoreactive cells was increased in hSOD1^G93A ^mice, but it was reduced by treatment with BV (E, F). Scale bars = 100 μm (C-F). Cell counting for TNF-α immunoreactive cells in saline- (black columns, n = 3) or BV-treated (white columns, n = 4) hSOD1^G93A ^mice (G, H). BV treatment reduced significantly TNF-a immunoreactivity in the brainstem (G) and lumbar spinal cord (H). Data were analyzed with a *t*-test. *p < 0.001. AH: anterior horn

In order to determine whether BV suppressed neuroinflammation by inhibiting of the release of the pro-inflammatory cytokine TNF-α, we further examined the level of TNF-α by immunohistochemistry in BV- or saline-treated familial ALS mice. As expected, TNF-α immunoreactivity in hSOD1^G93A ^mice was largely confined to the facial nucleus of the brain stem and motor neurons in the anterior horn of the spinal cord (Figure 5A,C, and 5E). Interestingly, BV caused a significant 4-fold reduction in TNF-α immunoreactivity in both the brainstem and lumbar spinal cord (Figure 5B,D, and 5F-H). These results suggest that BV treatment may be involved in an anti-neuroinflammatory responses that reduces motor neuron degeneration and prolongs the life span of hSOD1^G93A ^transgenic mice at the symptomatic stage.

### BV inhibits cell death and disruption of mitochondrial structure in hSOD1^G93A ^mice

**Figure 6:**
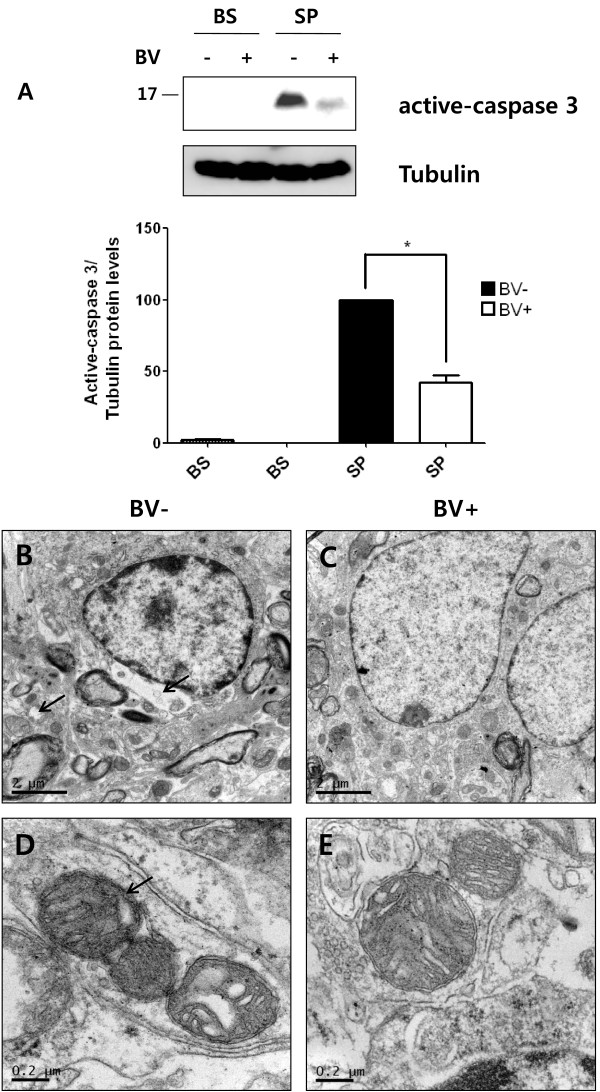
**The effect of BV on mitochondrial dysfunction in symptomatic mutant hSOD1**^**G93A **^**mice**. A representative of Western blotting analysis of the brainstem and lumbar spinal cord of BV- or saline-treated hSOD1^G93A ^mice (n = 3) using anti-active caspase-3 antibodies (A). The image is representative of three independent experiments. The optical density was measured for each band, and values for Iba 1 were compared with tubulin after correcting for the total protein content. The Results of the densitometric quantifications are the means ± SEM of triplicate samples. The data were analyzed using a *t*-test. *p < 0.01 versus the corresponding saline-treated group. The expression level of active caspase-3 protein in the lumbar spinal cord was dramatically reduced by treatment with BV in symptomatic hSOD1^G93A ^transgenic mice. Transmission electron microscopy (TEM) of mitochondria in the lumbar spinal cord of saline- or BV-treated symptomatic hSOD1^G93A ^mice (B-E). In symptomatic hSOD1^G93A ^mice, mitochondria displayed vacuolation (arrow, B) and broken cristae (arrow, D). However, BV treatment prohibited collapse of the mitochondrial structure and loss of cristae in familial symptomatic hSOD1^G93A ^mice (C, E). BV: bee venom, BS: brainstem, SP: spinal cord

The caspase family plays an important role in the pathogenesis of CNS disorders [24]. Caspase-3 activation, which has been detected in ALS patients [25], and mutant SOD1 expression have been shown to induce caspase-dependent neuronal apoptosis *in vitro *[26]. To assess whether BV affected mitochondrial cell death in symptomatic mutant SOD1 mice, we performed immunoblotting analysis of homogenates of the spinal cord and brainstem using a caspase-3 antibody. Expression levels of the active caspase-3 fragment were markedly increased in the lumbar spinal cord of hSOD1^G93A ^mice, but caspase-3 expression was maintained at a very low level in the spinal cord of BV-treated hSOD1^G93A ^mice. More specifically, caspase-3 expression was found to be reduced by 80% relative to the level observed in untreated hSOD1^G93A ^mice (Figure 6A).

Next, we asked whether BV affected the mitochondrial ultrastructure in the lumbar spinal cord of symptomatic hSOD1^G93A ^mice. We used transmission electron microscopy (TEM) to visualize mitochondria from the anterior horn of the lumbar spinal cord from BV- or saline-treated mutant SOD1^G93A ^mice. In symptomatic familial hSOD1^G93A ^mice, mitochondria displayed vacuolation and broken cristae (Figure 6B,D). By contrast, mitochondrial cristae were shaped as compact tubules in an orderly fashion in the ventral horn of the spinal cord from BV-treated (Figure 6C,E). These studies demonstrated that BV serves a protective role in regulating mitochondrial structure and cristae morphology.

**Figure 7:**
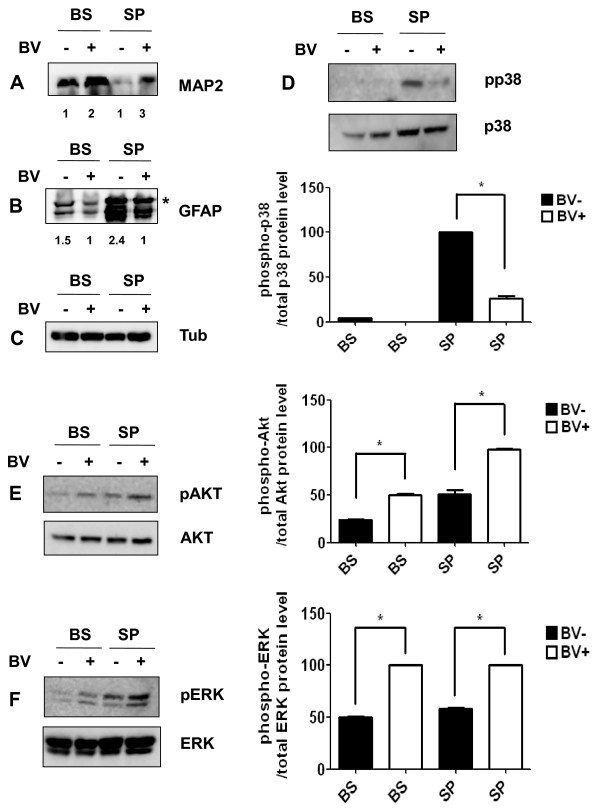
**BV treatment reduces the expression of GFAP protein and prevents neuronal cell death via modulation of cell survival signaling pathways**. A representative of Western blot analysis shows increased expression of the MAP protein (A), which is reduced in BV-treated hSOD1^G93A ^mice at the symptomatic stage (B). The intensities of the immunoreactive bands were compared to the corresponding bands from the brainstem or spinal cord of saline-treated mice (A). Quantification of the intensity of the immunoreactive band was compared to the corresponding bands from the brainstem or spinal cord of BV-treated hSOD1^G93A ^mice (n = 3) (B). * indicates a non-specific immunoblot band. Western blots show that the expression of active p38 in the spinal cord was reduced by BV treatment compared to the levels observed in age-matched hSOD1^G93A ^mice (n = 3) (D). The expression level of phospho-Akt protein was increased in the brain stem and lumbar spinal cord by BV treatment (E). The amount of phospho-ERK protein was augmented after BV treatment in tissue extracts from the brainstem and spinal cord of hSDO1^G93A ^mice (F). Quantitative analysis of immunoblot. The image is representative of three independent experiments. The optical density was measured for each band, and values for Iba-1 were compared with tubulin after correcting for the total protein content. The Results of the densitometric quantifications are the means ± SEM of triplicate samples. The data were analyzed using a *t*-test. *p < 0.01 versus the corresponding saline-treated group. BV: bee venom, BS: brainstem, SP: spinal cord

Next, we asked whether BV-induced signal transduction pathways affected neuron death and gliosis in hSOD1^G93A ^mice. As shown in Figure 7A, Western bolt analysis revealed an increase in the expression of the neuronal cell marker MAP2 in both the brainstem and lumbar spinal cord of BV-treated symptomatic hSOD1^G93A ^mice. In addition, GFAP was significantly reduced in the BV-treated brainstem and spinal cord when compared with age-matched familial mutant SOD1 mice (Figure 7B). These data suggest that BV-treatment at ST36 had a neuroprotective effect via activation of a cell survival signal transduction pathway, which reduced ALS-associated motor neuron death from gliosis and neuroinflammation.

### The effect of BV on cell survival signal transduction pathways

To evaluate the mechanism by which BV mediates this neuroprotective activity, we examined the expression levels of phospho-p38 MAPK and several anti-apoptotic markers, such as phospho-Akt and phospho-ERK, in symptomatic hSOD1^G93A ^mice. Western blotting experiments using anti-phospho-p38 or Ser435-phospho-specific Akt1 antibodies demonstrated activation of Akt-1 and deactivation of phospho-p38 in the spinal cord and brainstem following treatment with BV (Figure 7D,E). Furthermore, BV-treatment dramatically increased the expression of phospho-ERK in the spinal cord and brainstem of familial mutant SOD1 mice (Figure 7F). These biochemical results support that previous observations indicating that BV at ST36 improved motor activity and increased survival rates of hSOD1^G93A ^mice (Figures 2 and 3).

## Discussion

The molecular targets and signaling pathways influencing paralysis in ALS are not completely understood. To date, several pathogenic mechanisms have been identified that contribute to atrophy and paralysis in ALS patients, including protein misfolding, mitochondrial dysfunction, oxidative damage, cytoskeletal abnormalities, defective axonal transport, glutamate excitotoxicity, inadequate growth factor signaling, and neuroinflammation.

In this study, we demonstrated that administration of BV at a symptomatic stage of disease progression resulted in increased motor activity and a prolonged life span in comparison with age-matched control mice. Furthermore, we observed that BV prevented neuroinflammation-induced death of motor neurons and alleviated mitochondrial disruption in symptomatic SOD1^G93A ^transgenic mice.

Microglia in the CNS are immunologically active and capable of responding to events associated with formation of the neuronal-glial environment. Moreover, evidence indicating that microglial cells are involved in the progression of ALS pathogenesis has emerged from several lines of investigation. For example, activated microglia are typically found in ALS patients and in mouse models of ALS [27]. In addition, microglia have been shown to increase pro-inflammatory cytokines, reactive oxygen species (ROS), and glutamate in response to stimuli from neurons and astrocytes, which include pro-inflammatory cytokines and neurotrophic factors [28].

The pro-inflammatory cytokine TNF-α is a member of the TNF superfamily, which is a class of cytokines known to promote inflammatory signaling [29]. TNF signaling occurs through many factors including intracellular signaling pathways, nuclear factor kappa-B (NF-kB), p38, and c-Jun N-terminal kinase (JNK), which results in a number of responses such as inflammation, proliferation, cell migration, and necrosis [30,31]. TNF signaling has been shown to have several important functions within the CNS [32], including injury-mediated microglial and astrocyte activation [33]. Elevated levels of TNF-α and activation of TNF-α-mediated signaling pathways are evident in a large number of neurological disorders including AD, PD, and ALS [13-15]. In addition, cytokines TNF-α has been proposed to be involved in ALS-like microglial activation [16], and inflammation by this cytokine has been shown to induce motor neuron death [17]. In accordance with previous reports [27,15], the present study showed that microglial cell activation and increased TNF-α cytokine levels were involved in ALS pathologies involving both the spinal cord and the facial nucleus of the brainstem in symptomatic familial hSOD1^G93A ^mice (Figures 4, 5). Interestingly, we found that BV administration at the ST36 acupoint significantly attenuated neuroinflammatory events triggered by TNF-α and microglial cell activation in motor function related-structures within the spinal cord and brainstem (Figures 4A, 5G-H). Some reports have also suggested that BV may be an efficient treatment for multiple sclerosis (MS) and other autoimmune diseases based on the inhibition of TNF-α production [34,35,18]. These findings suggest that BV treatment may be useful for combating inflammation in ALS patients.

Previous reports have indicated that a common mechanism of toxicity associated with the mutant form of SOD1 is the sequential activation of caspase-1 and caspase-3 [36]. Since caspase-3 activation leads to microglial cell activation and mitochondrial dysfunction, we examined whether caspase-3 inactivation was involved in the anti-inflammatory effect of BV. We found that the levels of active caspase-3 were significantly reduced in the lumbar spinal cord of hSOD1^G93A ^mice following BV treatment (Figure 6A). Furthermore, the administration of BV prevented the disruption of mitochondrial cristae and vacuolization in the ventral horn of hSOD1^G93A ^mice at the symptomatic stage (Figure 6B-E). This result suggests that BV treatment had an anti-inflammatory effect on the CNS of familial mutant SOD1 mice. Thus, we hypothesized that BV-treatment reduced motor neuron death and mitochondrial dysfunction by preventing neuroinflammation. Supporting this idea, BV treatment significantly increased the expression of a neuronal cell marker and reduced GFAP levels when compared with age-matched hSOD1^G93A ^transgenic mice (Figure 7A-B). Accordingly, a reduced number of astrocytes and diminished cytokine levels positively influenced neuronal survival and inhibited mitochondrial dysfunction which otherwise might have led to increased levels of reactive oxygen species (ROS) and glutamate release associated with ALS [37]. With respect to the molecular mechanism underlying BV, we observed that BV treatment triggered a reduction in the activation of p38 MAPK, which is downstream of the TNF-α signaling pathway in the spinal cord of hSOD1mice (Figure 7D). In contrast, the levels of phospho-AKT and phospho-ERK were increased in both the brainstem and spinal cord of BV-treated hSOD1 mice compared to those of control mice (Figure 7E-F). These results demonstrate that BV-treatment of familial mutant hSOD1 transgenic mice caused a reduction in pro-inflammatory cytokines and an increase in phospho-Akt and ERK, which may inhibit motor neuronal cell death by preventing neuroninflammation and consequently delay disease onset.

## Conclusions

This study presented that the improved motor activity and prolonged life span of BV-treated hSOD1^G93A ^mice were attributable to the neuroprotective effect provide by reduced levels of cytokines, which are typically released by activated microglia and astrocytes. Furthermore, the present study illustrated that BV treatment prevented mitochondrial disruption and served a neuroprotective role *in vivo *via the activation of cell survival signal transduction pathways, such as the PI3K and ERK pathways, which subsequently protected against the death of motor neurons in symptomatic hSOD1^G93A ^mice.

Further challenges remaining will be to determine whether bee venom treatment at other acupoints presents neuro-protective effects against neuroinflammation in symptomatic ALS mice and find the potential bioactive element of bee venom components *in vivo *and *in vitro*.

## Abbreviations

ALS: Amyotrophic lateral sclerosis; CNS: central nervous system; mtSOD1: mutant SOD1; ST36: Zusanli; fALS: Familial ALS; hSOD1: human Cu-Zn superoxide dismutase 1; TNF-α: tumor necrosis factor-alpha; AD: Alzheimer's disease; IL-1: interleukin-1; NOS: nitric oxide synthetase; MS: multiple sclerosis; PD: Parkinson's disease; IFN-γ: interferon-gamma; BV: Bee venom; MAPK: mitogen-activated protein kinase

## Completing interests

The authors declare that they have no competing interests.

## Authors' contributions

EJY designed the experiments and analyzed the data as well as wrote the manuscript. JHJ carried out the rotarod test, immunohistochemistry and performed statistical analyses. SML and HSH participated in the tissue processing of animal for all experiments. SCY contributed technical expertise for TEM. MSL and SMC helped editing of the manuscript. All authors have read and approved the final manuscript. All data collection and observations were completed by a blinded observer.

## References

[B1] Mulder DW Clinical limits of amyotrophic lateral sclerosis Adv Neurol 1982 36 15 22 7180681

[B2] Cleveland DW Rothstein JD From Charcot to Lou Gehrig: deciphering selective motor neuron death in ALS Nat Rev Neurosci 2001 2 806 819 10.1038/35097565 11715057

[B3] Weishaupt JH Bartels C Polking E Dietrich J Rohde G Poeggeler B Mertens N Sperling S Bohn M Huther G Schneider A Bach A Siren AL Hardeland R Bahr M Nave KA Ehrenreich H Reduced oxidative damage in ALS by high-dose enteral melatonin treatment J Pineal Res 2006 41 313 323 10.1111/j.1600-079X.2006.00377.x 17014688

[B4] Rosen DR Siddique T Patterson D Figlewicz DA Sapp P Hentati A Donaldson D Goto J O'Regan JP Deng HX Rahmani Z Krizus A McKenna-Yasek D Cayabyab A Gaston SM Berger R Tanzi RE Halperin JJ Herzfeldt B Bergh RV Hung W Bird T Deng G Mulder DW Smyth C Laing NG Soriano E Pericak-Vance MA Haines J Rouleau GA Gusella JS Horvitz HR Brown RH Jr Mutations in Cu/Zn superoxide dismutase gene are associated with familial amyotrophic lateral sclerosis Nature 1993 362 59 62 10.1038/362059a0 8446170

[B5] Gurney ME Pu H Chiu AY Dal Canto MC Polchow CY Alexander DD Caliendo J Hentati A Kwon YW Deng HX Motor neuron degeneration in mice that express a human Cu, Zn superoxide dismutase mutation Science 1994 264 1772 1775 10.1126/science.8209258 8209258

[B6] Brown RH Brown RH Robberecht W Amyotrophic lateral sclerosis: pathogenesis Semin Neurol 2001 21 131 139 10.1055/s-2001-15260 11442322

[B7] Pasinelli P Brown RH Molecular biology of amyotrophic lateral sclerosis: insights from genetics Nat Rev Neurosci 2006 7 710 723 10.1038/nrn1971 16924260

[B8] McGeer PL McGeer EG Inflammatory processes in amyotrophic lateral sclerosis Muscle Nerve 2002 26 459 470 10.1002/mus.10191 12362410

[B9] Weydt P Weydt P Yuen EC Ransom BR Moller T Increased cytotoxic potential of microglia from ALS-transgenic mice Glia 2004 48 179 182 10.1002/glia.20062 15378658

[B10] Streit WJ Mrak RE Griffin WS Microglia and neuroinflammation: a pathological perspective J Neuroinflammation 2004 1 14 10.1186/1742-2094-1-14 15285801 PMC509427

[B11] Schindler JF Monahan JB Smith WG p38 pathway kinases as anti-inflammatory drug targets J Dent Res 2007 86 800 811 10.1177/154405910708600902 17720847

[B12] Hofman FM Hinton DR Johnson K Merrill JE Tumor necrosis factor identified in multiple sclerosis brain J Exp Med 1989 170 607 612 10.1084/jem.170.2.607 2754393 PMC2189402

[B13] Fillit H Ding WH Buee L Kalman J Altstiel L Lawlor B Wolf-Klein G Elevated circulating tumor necrosis factor levels in Alzheimer's disease Neurosci Lett 1991 129 318 320 10.1016/0304-3940(91)90490-K 1745413

[B14] Hirsch EC Hunot S Damier P Faucheux B Glial cells and inflammation in Parkinson's disease: a role in neurodegeneration? Ann Neurol 1998 44 S115 S120 9749582 10.1002/ana.410440717

[B15] Poloni M Facchetti D Mai R Micheli A Agnoletti L Francolini G Mora G Camana C Mazzini L Bachetti T Circulating levels of tumor necrosis factor-alpha and its soluble receptors are increased in the blood of patients with amyotrophic lateral sclerosis Neurosci Lett 2000 287 211 214 10.1016/S0304-3940(00)01177-0 10863032

[B16] Wen W Sanelli T Ge W Strong W Strong MJ Activated microglial supernatant induced motor neuron cytotoxicity is associated with upregulation of the TNFR1 receptor Neurosci Res 2006 55 87 95 10.1016/j.neures.2006.02.004 16529832

[B17] Mir M Asensio VJ Tolosa L Gou-Fabregas M Soler RM Llado J Olmos G Tumor necrosis factor alpha and interferon gamma cooperatively induce oxidative stress and motoneuron death in rat spinal cord embryonic explants Neuroscience 2009 162 959 971 10.1016/j.neuroscience.2009.05.049 19477238

[B18] Somerfield SD Brandwein S Bee venom and adjuvant arthritis J Rheumatol 1988 15 1878 3230576

[B19] Wilms H Zecca L Rosenstiel P Sievers J Deuschl G Lucius R Inflammation in Parkinson's diseases and other neurodegenerative diseases: cause and therapeutic implications Curr Pharm Des 2007 13 1925 1928 10.2174/138161207780858429 17584117

[B20] Yin CS Jeong HS Park HJ Baik Y Yoon MH Choi CB Koh HG A proposed transpositional acupoint system in a mouse and rat model Res Vet Sci 2008 842 159 165 10.1016/j.rvsc.2007.04.004 17559895

[B21] Azzouz M Ralph GS Storkebaum E Walmsley LE Mitrophanous KA Kingsman SM Carmeliet P Mazarakis ND VEGF delivery with retrogradely transported lentivector prolongs survival in a mouse ALS model Nature 2004 429 413 417 10.1038/nature02544 15164063

[B22] Kirkinezos IG Bacman SR Hernandez D Oca-Cossio J Arias LJ Perez-Pinzon MA Bradley WG Moraes CT Cytochrome c association with the inner mitochondrial membrane is impaired in the CNS of G93A-SOD1 mice J Neurosci 2005 25 164 172 10.1523/JNEUROSCI.3829-04.2005 15634778 PMC6725219

[B23] Wyss-Coray T Mucke L Inflammation in neurodegenerative disease--a double-edged sword Neuron 2002 35 419 432 10.1016/S0896-6273(02)00794-8 12165466

[B24] Hara H Friedlander RM Gagliardini V Ayata C Fink K Huang Z Shimizu-Sasamata M Yuan J Moskowitz MA Inhibition of interleukin 1beta converting enzyme family proteases reduces ischemic and excitotoxic neuronal damage Proc Natl Acad Sci USA 1997 94 2007 2012 10.1073/pnas.94.5.2007 9050895 PMC20033

[B25] Martin LJ Price AC Kaiser A Shaikh AY Liu Z Mechanisms for neuronal degeneration in amyotrophic lateral sclerosis and in models of motor neuron death (Review) Int J Mol Med 2000 5 3 13 10601567 10.3892/ijmm.5.1.3

[B26] Troy CM Stefanis L Prochiantz A Greene LA Shelanski ML The contrasting roles of ICE family proteases and interleukin-1beta in apoptosis induced by trophic factor withdrawal and by copper/zinc superoxide dismutase down-regulation Proc Natl Acad Sci USA 1996 93 5635 5640 10.1073/pnas.93.11.5635 8643629 PMC39300

[B27] Boillee S Vande Velde C Cleveland DW ALS: a disease of motor neurons and their nonneuronal neighbors Neuron 2006 52 39 59 10.1016/j.neuron.2006.09.018 17015226

[B28] Moisse K Strong MJ Innate immunity in amyotrophic lateral sclerosis Biochim Biophys Acta 2006 1762 1083 1093 16624536 10.1016/j.bbadis.2006.03.001

[B29] Wajant H Pfizenmaier K Scheurich P Tumor necrosis factor signaling Cell Death Differ 2003 10 45 65 10.1038/sj.cdd.4401189 12655295

[B30] Ware CF Network communications: lymphotoxins, LIGHT, and TNF Annu Rev Immunol 2005 23 787 819 10.1146/annurev.immunol.23.021704.115719 15771586

[B31] Eissner G Kolch W Scheurich P Ligands working as receptors: reverse signaling by members of the TNF superfamily enhance the plasticity of the immune system Cytokine Growth Factor Rev 2004 15 353 366 10.1016/j.cytogfr.2004.03.011 15450251

[B32] Tansey MG Wyss-Coray T Lane TE, Carson M, Bergmann C, Wyss-Coray T Cytokines in CNS inflammation and disease Central Nervous System Diseases and Inflammation 2008 California: Springer 59 106 full_text

[B33] Selmaj KW Farooq M Norton WT Raine CS Brosnan CF Proliferation of astrocytes in vitro in response to cytokines: A primary role for tumor necrosis factor J Immunol 1990 144 129 135 2104886

[B34] Castro HJ Mendez-Lnocencio JI Omidvar B Omidvar J Santilli J Nielsen HS Jr Pavot AP Richert JR Bellanti JA A phase I study of the safety of honeybee venom extract as a possible treatment for patients with progressive forms of multiple sclerosis Allergy Asthma Proc 2005 26 470 476 16541972

[B35] Nam KW Je KH Lee JH Han HJ Lee HJ Kang SK Mar W Inhibition of COX-2 activity and proinflammatory cytokines (TNF-alpha and IL-1beta) production by water-soluble sub-fractionated parts from bee (Apis mellifera) venom Arch Pharm Res 2003 26 383 388 10.1007/BF02976695 12785734

[B36] Pasinelli P Houseweart MK Brown RH Cleveland DW Caspase-1 and -3 are sequentially activated in motor neuron death in Cu,Zn superoxide dismutase-mediated familial amyotrophic lateral sclerosis Proc Natl Acad Sci USA 2000 97 13901 13906 10.1073/pnas.240305897 11095709 PMC17673

[B37] Nagai M Re DB Nagata T Chalazonitis A Jessell TM Wichterle H Przedborski S Astrocytes expressing ALS-linked mutated SOD1 release factors selectively toxic to motor neurons Nat Neurosci 2007 10 615 622 10.1038/nn1876 17435755 PMC3799799

